# Appropriate preoperative membranous urethral length predicts recovery of urinary continence after robot-assisted laparoscopic prostatectomy

**DOI:** 10.1186/s12957-018-1523-2

**Published:** 2018-11-16

**Authors:** Daiki Ikarashi, Yoichiro Kato, Mitsugu Kanehira, Ryo Takata, Akito Ito, Mitsutaka Onoda, Renpei Kato, Tomohiko Matsuura, Kazuhiro Iwasaki, Wataru Obara

**Affiliations:** Department of Urology, Iwate Medical University School of Medicine, 19-1, Uchimaru, Morioka-shi, Iwate, 020-8505 Japan

**Keywords:** Membranous urethral length, Urinary continence, Robot-assisted laparoscopic prostatectomy

## Abstract

**Purpose:**

We investigated that preoperative membranous urethral length (MUL) would be associated with the recovery of urinary continence after robot-assisted laparoscopic prostatectomy (RALP).

**Patients and methods:**

We studied 204 patients who underwent RALP between May 2013 and March 2016. All patients underwent pelvic magnetic resonance imaging (MRI) preoperatively to measure MUL. Urinary continence was defined as the use of one pad or less (safety pad). The 204 patients were divided into two groups: continence group, those who achieved recovery of continence at 3, 6, and 12 months after RALP, and incontinence group, those who did not. We retrospectively analyzed the patients in terms of preoperative clinical factors including age, body mass index (BMI), estimated prostate volume, neurovascular bundle salvage, history of preoperative hormonal therapy, and MUL.

**Results:**

The safety pad use rate was 69.6%, 86.9%, and 91.1% at 3, 6, and 12 months, respectively. On univariate and multivariate analyses, MUL were significant factors in every term of recovery of urinary continence in both groups. According to the receiver operating characteristic (ROC) curve analysis, the preoperative MUL that could best predict early recovery of urinary continence at 3 months after RALP was 12 mm.

**Conclusions:**

We suggest that preoperative MUL > 12 mm would be a predictor of early recovery of urinary continence after RALP.

## Introduction

Urinary incontinence is one of the most unfavorable complications influencing the quality of life for patients after radical prostatectomy (RP). In early 2000, the initial robot-assisted laparoscopic prostatectomy (RALP) was performed with the da Vinci surgical system [1]. Currently, RALP has become a more popular surgical procedure for RP in Japan. RALP is expected to achieve better outcomes regarding recovery of urinary continence than did the conventional procedure. Its advanced technology provides a three-dimensional operative view and laparoscopic instruments that mimic the movement of the human wrist. For the robotic approach, a meta-analysis of 51 studies showed statistically significant improvement in urinary continence recovery at 12 months with RALP compared to retropubic and laparoscopic RP [2].

Predictive factors for recovery of urinary continence after RP, such as patient age, body mass index (BMI), and prostate volume, have been reported [2, 3]. Membranous urethral length (MUL) as measured by magnetic resonance imaging (MRI) also was reported to be a strong predictive factor for recovery of urinary continence in a systematic review and meta-analysis [4]. These reports demonstrated that preoperative MUL is associated significantly and positively with a return to continence following RP. However, few reports exist on the association of MUL and recovery of urinary continence after RALP in the Japanese population.

We evaluated the association of preoperative MUL with the recovery of urinary incontinence after RALP in Japanese patients.

## Patients and methods

We performed RALP using the da Vinci Si surgical system in 204 consecutive patients from whom we could collect the Expanded Prostate Cancer Index Composite (EPIC) questionnaire [5] at least preoperatively and 3 months after RALP between May 2013 and March 2016. All patients underwent pelvic MRI preoperatively. Most patients had no urinary incontinence before surgery; only ten patients had incontinence more than once a week. All surgical procedures were performed by four surgeons (YK, MK, RT, and WO). All surgeons are over 10 years as urologists, and each surgeon experienced RALP in more than 40 cases. RALP was performed via the conventional transperitoneal approach using the four-armed da Vinci surgical robot system [6]. In all cases, we performed the Rocco technique for posterior reconstruction of Denonvillier’s facia [7], anterior preservation [8], and bladder neck preservation [9] for preventing incontinence. Hemi–nerve sparing was performed depending on the cancer status [10]. Pelvic lymph node dissection also was performed in patients with a high risk of cancer according to the D’Amico criteria. We also offered all patients pelvic floor exercise education during the operative period.

**Fig. 1:**
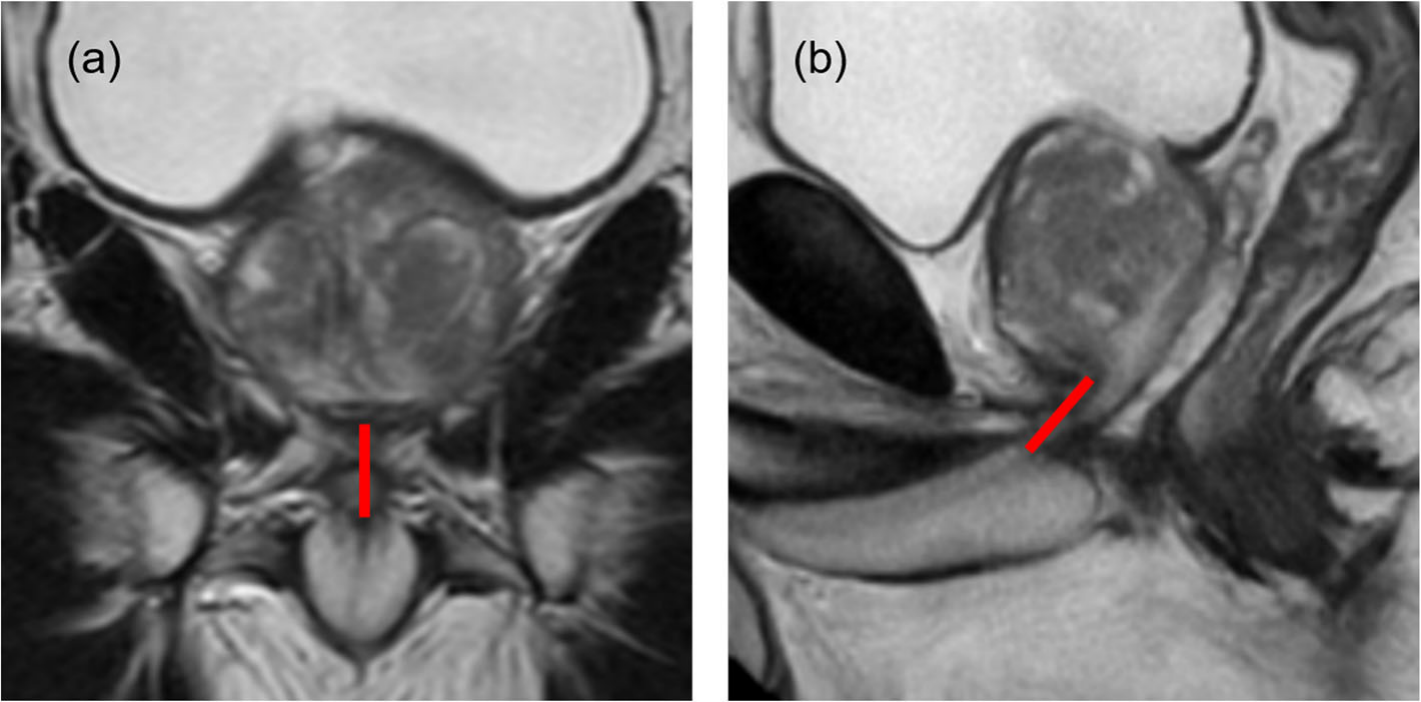
The preoperative membranous urethral length as the distance from the prostatic apex to the level of the urethra at the penile bulb is measured in the T2-weighted MRI **a** coronal and **b** sagittal planes

The MUL was measured by T2-weighted coronal and sagittal sections as a distance from the prostatic apex to the level of the urethra at the penile bulb on preoperative pelvic MRI (Fig 1) [11]. In terms of measuring the MUL, a number of urologists evaluated MUL of each case at the preoperative conference. Thereafter, the data of MUL was remeasured by a researcher.

Urinary continence was evaluated at 3, 6, and 12 months after RALP using question 5 of the EPIC questionnaire [5]. Continence was defined as the use of one pad or less as a safety pad. We also examined the quality of life (QOL) score regarding urinary continence pre- and postoperatively using question 12 of the EPIC questionnaire [5].

The patients were divided into two groups: continence group, those who achieved recovery of urinary continence within 3, 6, 12 months, and incontinence group, those who did not.

Univariate analysis was performed with the *t* test, analysis of variance, chi-square, and Fisher’s exact test between continence and incontinence group regarding preoperative clinical factors including patient age, BMI, estimated prostate volume, clinical stage, neurovascular bundle salvage, history of preoperative hormonal therapy, positive surgical margin, leakage at vesicourethral anastomosis, and MUL. Multivariate analysis was performed using a logistic regression model, and significances were tested using a likelihood ratio test. Statistical analyses were performed using JMP software (SAS Institute, Inc., Cary, NC, USA). For all statistical comparisons, differences with *P* < 0.05 were considered statistically significant.

## Result

**Fig. 2:**
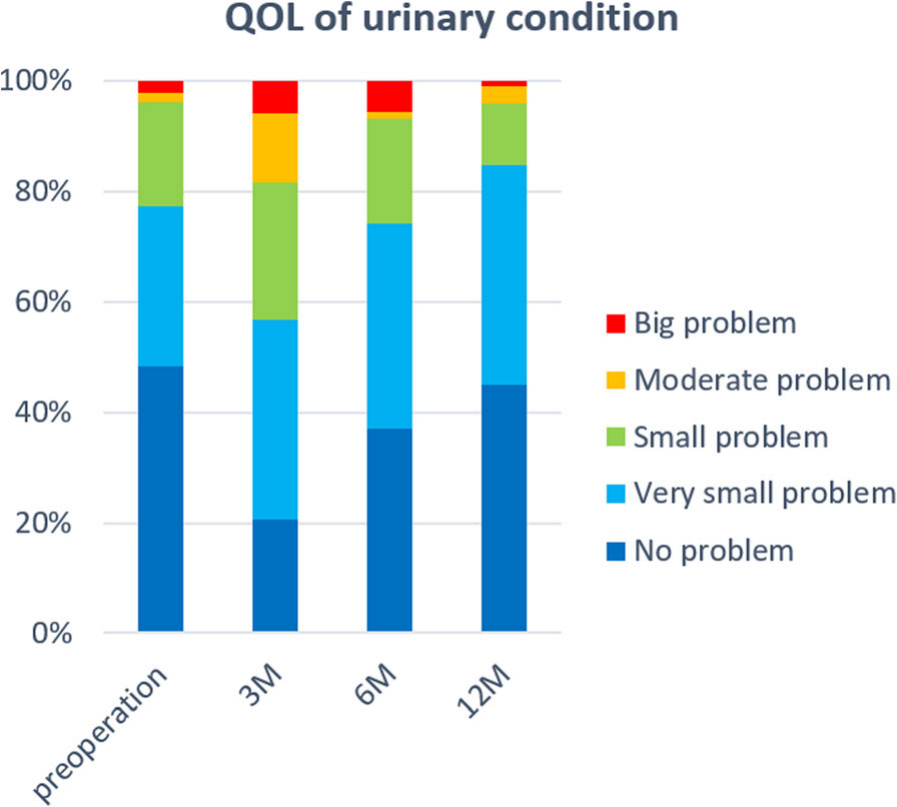
QOL score of urinary condition per time period after RALP. The QOL status after RALP had the worst at 3 months. It was gradually improved, and 1 year after surgery was almost improved

Mean patient age was 65 years, BMI was 23.7 kg/m^2^, prostate-specific antigen (PSA) was 6.5 ng/ml, and MUL was 13.1 mm. Preoperative hormonal therapy was given in 34 (16.7%) patients, and hemi–nerve sparing was performed in 70 (34.6%; Table 1). The safety pad rate was 69.6%, 86.9%, and 91.1% at 3, 6, and 12 months, respectively, and the pad-free rate was 33.8%, 49.7%, and 64.3%, respectively. The QOL regarding the urinary condition after RALP was worst at 3 months. It improved gradually, and at 1 year postoperatively, it had improved to the preoperative status (Fig. 2).

**Table 1:** Patient characteristics

Characteristic	Total (*n* = 204)
Median (range)	
Observation period, days	350 (82–545)
Age, years	65 (41–76)
BMI, kg/m^2^	23.7 (17.2–37.6)
PSA level, ng/ml	6.5(3.5–46.4)
Preoperative MUL, mm	13.1 (4.5–22.9)
Estimated prostate volume, g	38 (7–94)
Console time, min	143 (87–351)
Operation time without console, min	52 (18–93)
Intraoperative bleeding, ml*	70 (10–1243)
*N* (%)	
Clinical stage	
^T2a	159 (77.9%)
T2b	24 (11.8%)
^T2c	21 (10.3%)
Preoperative hormonal therapy history	34 (16.7%)
Lymph node dissection	58 (28.4%)
Neurovascular bundle saving	70 (34.6%)
Positive surgical margin	59 (28.9%)
Leakage at the vesicourethral anastomosis	17 (8.3%)

*Including urine

**Fig. 3:**
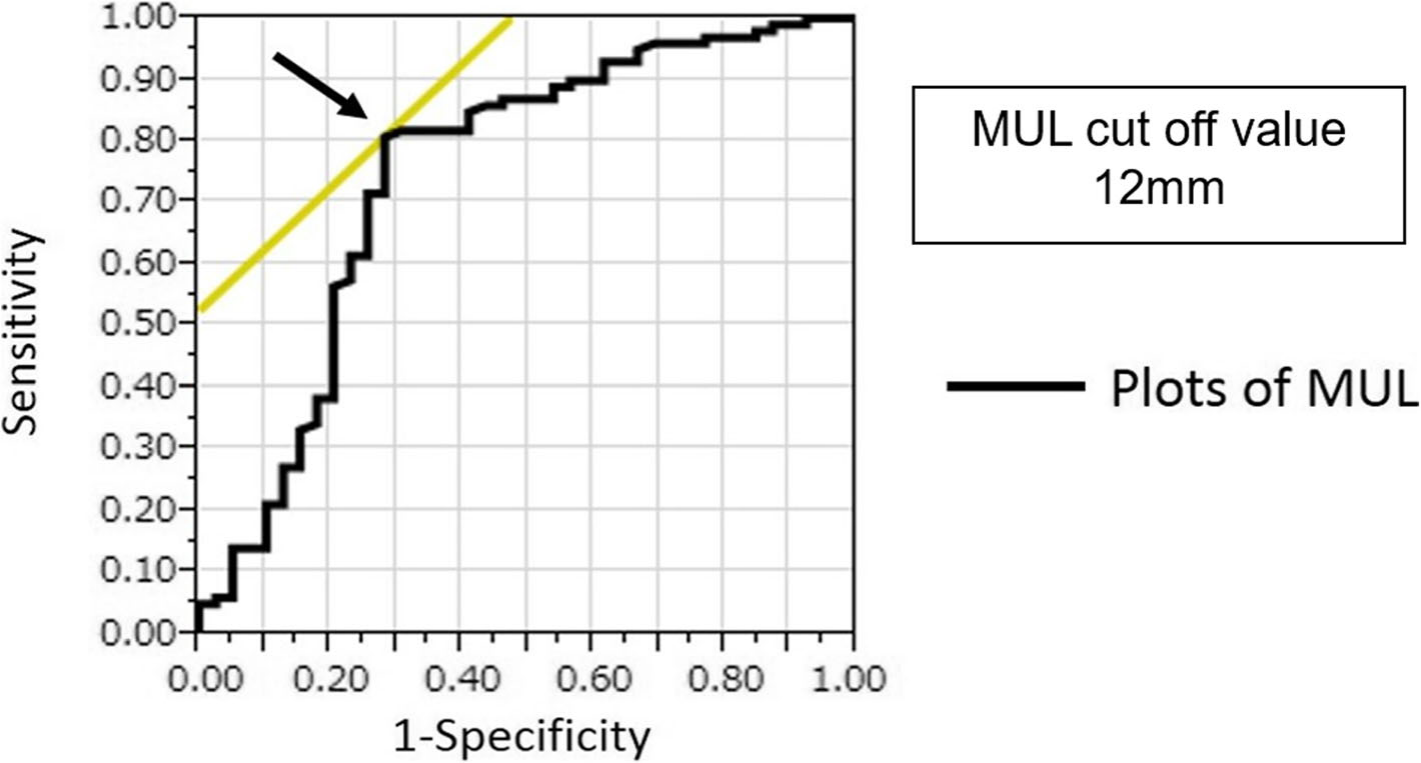
ROC curve for recovery of urinary continence 3 months after RALP. The cutoff point of MUL in 12 mm was clearly classified into continence group and incontinence group

In the univariate analysis for recovery of urinary continence 3 months after RALP, patient age (*P* = 0.034), and MUL (*P* < 0.001) were statistically significantly associated with safety pad use. At 6 months after RALP, MUL (*P* = 0.004), console time, and leakage at vesicourethral anastomosis were statistically significant. At 12 months after RALP, MUL (*P* = 0.023) was statistically significant (Table 2). On multivariate analysis, patient age, leakage at vesicourethral anastomosis, and MUL achieved statistical significance with safety pad use. Among them, MUL was the most statistically significant with safety pad at 3 months after RALP (Table 3). Moreover, we estimated the optimal length of the MUL that could best classify between the continence and incontinence group. The cutoff point of MUL for recovery of urinary continence at 3 months after RALP was determined using the receiver operating characteristic (ROC) analysis. We identified a reasonable cutoff point of MUL to be 12 mm (Fig. 3). MUL > 12 mm was a favorable predictor of recovery of urinary continence at 3 months after RALP. Furthermore, using the cutoff point of MUL 12 mm, our cases were classified into continence and incontinence groups with a sensitivity of 80% and specificity of 70% (Fig. 3).

**Table 2:** Univariate analysis

Parameters	Continence at 3 months (*n* = 204)			Continence at 6 months (*n* = 175)			Continence at 12 months (*n* = 112)		
	Continence (*n* = 142)	Incontinence (*n* = 62)	*P*	Continence (*n* = 152)	Incontinence (*n* = 23)	*P*	Continence (*n* = 102)	Incontinence (*n* = 10)	*P*
Median (range)									
Age, years	65 (41–74)	67 (51–76)	0.034	66 (58–76)	64 (41–75)	0.989	66 (50–76)	64 (58–73)	0.689
BMI, kg/m^2^	23.6 (17.2–37.6)	23.9 (18.9–33.1)	0.213	23.8 (17.2–37.6)	23.6 (18.9–33.1)	0.507	23.7 (17.2–32.7)	24.5 (18.9–33.1)	0.391
PSA level, ng/ml	6.7 (3.5–46.4)	5.6 (4.2–24.1)	0.178	6.4 (3.5–46.4)	6.6 (4.2–14.5)	0.833	6.2 (3.8–32.1)	6.8 (4.2–16.7)	0.669
Preoperative MUL, mm	13.6 (8.3–22.9)	11.1 (4.5–17.9)	< .001	13.5 (6.5–22.9)	11.1 (4.5–17.3)	0.004	13.4 (6.5–22.9)	10.8 (4.5–17.3)	0.023
Estimated prostate volume, g	38 (7–94)	38 (17–92)	0.481	38 (7–94)	41 (20–92)	0.119	39 (14–94)	49 (30–92)	0.758
Console time, min	140 (87–351)	150 (99–339)	0.062	139 (89–253)	157 (99–280)	0.028	135 (89–253)	177 (99–280)	0.059
Operation time without console, min	51 (18–93)	54 (23–86)	0.792	49 (18–93)	54 (23–86)	0.477	51 (24–93)	56 (23–86)	0.506
Intraoperative bleeding, ml	68 (10–1243)	83 (16–535)	0.759	66 (14–1243)	85 (20–397)	0.696	70 (14–1243)	78 (26–320)	0.967
*N* (%)									
Clinical stage			0.218			0.763			0.532
^T2a	109 (76.8%)	50 (80.6%)		119 (78.3%)	18 (78.2%)		83 (81.4%)	9 (90%)	
T2b	20 (14.1%)	4 (6.5%)		19 (12.5%)	2 (8.7%)		13 (12.7%)	1 (10%)	
^T2c	13 (9.1%)	8 (12.9%)		14 (9.2%)	3 (13.1%)		6 (5.9%)	0	
Preoperative hormonal therapy history	21 (14.8%)	13 (20.9%)	0.276	20 (13.2%)	5 (21.7%)	0.298	11 (10.8%)	3 (30%)	0.122
Lymph node dissection	40 (28.2%)	18 (29%)	0.901	43 (28.3%)	6 (26.1%)	0.825	25 (24.5%)	2 (20%)	0.746
Neurovascular bundle saving	52 (36.6%)	18 (29%)	0.312	57 (37.5%)	8 (34.8%)	0.901	39 (38.2%)	4 (40%)	0.951
Positive surgical margin	43 (30.3%)	17 (27.4%)	0.678	45 (29.6%)	4 (17.4%)	0.224	29 (28.4%)	1 (10%)	0.209
Leakage at vesicourethral anastomosis	9 (6.3%)	9 (14.5%)	0.068	8 (5.3%)	7 (30.4%)	0.007	5 (4.9%)	2 (20%)	0.059

*BMI* body mass index, *PSA* prostate-specific antigen, *MUL* membranous urethral length

**Table 3:** Multivariate analysis

Parameters	Continence at 3 months (*n* = 204)			Continence at 6 months (*n*=		175)	Continence at 12 months (*n* = 112)		
	OR	95%CI	*P*	OR	95%CI	*P*	OR	95%CI	*P*
Age, years	1.074	1.01–1.15	0.028		–			–	
Preoperative MUL, mm	0.635	0.53–0.74	< .0001	0.699	0.56–0.85	0.0002	0.743	0.56–0.96 0.026	
Console time, min		–		1.007	0.99–1.02	0.167	0.997	0.95–1.05 0.09	
Leakage at vesicourethral anastomosis		–		0.123	0.03–0.43	0.0014		–	

*OR* odds ratio, *CI* confidence interval

## Discussion

RALP is expected to affect not only cancer control but also functional outcomes, such as continence and potency [12]. Especially, many reviews reported that patients who underwent RALP would tend to recover urinary continence within 1 year postoperatively earlier than patients who underwent retropubic RP (RRP) [12–14]. Early recovery of urinary continence is one of the strongest points of RALP. However, some patients suffer severe urinary incontinence after RALP. Therefore, we conducted this study to investigate factors that influence recovery of urinary continence in patients undergoing RALP.

Several preoperative predictive factors for recovery of urinary continence after RALP, such as age, body mass index, prostate size, medical comorbidities, history of transurethral resection of the prostate (TURP), and history of preoperative urinary continence or lower urinary tract symptoms, were identified as studied previously. MUL based on preoperative imaging also is a predictive factor associated with urinary continence [15]. Coakley et al. [16] reported the first study using endorectal MRI to measure preoperative MUL and showed a correlation of MUL with urinary continence after RRP [13]. They demonstrated that a longer preoperative MUL was associated with a faster recovery of continence; at 1 year postoperatively, 120 of 134 patients (89%) with preoperative MUL > 12 mm were completely continent, compared to only 35 of 46 (76%) whose preoperative MUL was ≤ 12 mm. Despite the preoperative median length of MUL seems to be a minor difference among continence and incontinence groups in our study, in prior studies, an increase in MUL as low as 1 mm can increase the odds of return to continence up to 200% [17]. Our study demonstrated that preoperative MUL > 12 mm was the strongest factor indicating recovery of urinary continence 3 months after RALP. We identified 3 months after RALP as the early period for recovery of urinary continence because the result of QOL testing at 3 months after RALP was by no means satisfactory, and there still was room for improvement.

A previous study reported that MUL has anatomical variation [14]. Our study showed similar data as those reported in Korean patients [18, 19]. The average MUL in Asian patients may be approximately 12 mm. On the other hand, MUL in reports from the USA and Europe is slightly longer than that of Asians, but the racial difference is not clear because the number of reports is too small [15, 20].

MUL also has been associated with urinary continence recovery after RP [4, 15, 17–19]. Paparerl et al. [11] reported that a loss ratio of MUL between pre- and postoperatively was associated with postoperative incontinence. They suggested that preserving MUL is important for time-to-recovery and degree of recovery. The membranous urethra contains smooth muscle fibers along its entire length and is surrounded by the rhabdosphincter [21–23]. Decreasing intraoperative trauma when preserving the MUL, which includes a greater amount of smooth muscle fibers and rhabdosphincter, has an important role in continence after RP because it contributes to maintaining and increasing urethral closure pressure [24].

Our result suggested that a longer preoperative MUL has advantages for early urinary continence recovery after RALP. In the open era, MUL might not have been taken into account as an operator’s factor, but in the RALP era, the anatomical difference is evident more clearly and objectively. Therefore, not only the different operator technique, but also the MUL would relate directly to the early recovery of urinary continence.

In multivariate analysis, increasing patient age also was a risk factor for incontinence after RALP. Several studies showed a greater impact on urinary continence recovery with increasing age [25, 26]. Meanwhile, Basto et al. [27] reported urinary continence recovery rates after RALP in older men that were comparable to their younger counterparts, and thus, this should not be a reason to deny older men with a reasonable life-expectancy curative treatment for localized prostate cancer. In addition, leakage at vesicourethral anastomosis was one of the risk factors for incontinence after RALP in multivariate analysis. Leakage at vesicourethral anastomosis causes inflammatory change followed by fibrotic tissue development around the anastomotic site and sometimes causes a refractory fistula. Stavros et al. [28] reported leakage at vesicourethral anastomosis causes complications including incontinence after prostatectomy. When the MUL is < 12 mm, the patient is older or complication of leakage at vesicourethral anastomosis, early recovery of urination would not be easy. Therefore, we suggested that MUL is an important factor for preoperative informed consent regarding continence.

Our study has several limitations. First, there are some inherent limitations of pad use as an outcome measure. We defined continence as the use of one pad or less per day and did not measure pad weight. What is important in actual clinical situations is whether the number of pads is related to QOL improvement. Therefore, we investigated a QOL questionnaire for the use of a security pad. Second, MRI was performed before RALP, while MUL was measured retrospectively. In terms of measuring MUL, we evaluated MUL by a number of urologists at the preoperative conference. Therefore, the data of MUL which were summarized by a urologist who was blinded to clinical data would be more reproducible and for less selective bias in this study. Third, there was a negative impact between urinary continence and nerve sparing in our study. The aim of nerve preservation was not only for urinary continence but also for the prevention of erectile dysfunction. We also considered that cancer control was more important. Therefore, we have performed nerve preservation on one side where cancer was not detected. There was no case of bilateral nerve preservation. Although 70 cases were performed on nerve preservation, no significant difference in urinary incontinence rate between preservation group and no preservation group was shown (*p* = 0.312). Steineck et al. [10] reported that there is a relation between nerve preservation and urinary incontinence, but their study examined nerve preservation on both sides. In contrast, Pick et al. [29] reported no significant difference was found in continence rates after RALP between hemi–nerve sparing and no nerve sparing. We consider that a more detailed examination about an association between nerve sparing and urinary incontinence is necessary. Finally, the retrospective design also might be a limitation. We currently are performing a prospective examination of a modified procedure on the prostatic apex according to the MUL during RALP.

## Conclusion

In conclusion, preoperative MUL > 12 mm would be a predictive factor for the recovery of urinary continence at 3 months after RALP. Evaluation of preoperative MUL would be useful in clinical settings because it is easy to measure and to acquire beneficial informed consent. This result should be validated by well-conducted prospective randomized controlled trials in the future.

## Abbreviations

BMI: Body mass index
EPIC: Expanded Prostate Cancer Index Composite
MRI: Magnetic resonance imaging
MUL: Membranous urethral length
PSA: Prostate-specific antigen
QOL: Quality of life
RALP: Robot-assisted laparoscopic prostatectomy
ROC: Receiver operating characteristic
RP: Radical prostatectomy
TURP: Transurethral resection of the prostate
